# Outcome of gastric emptying and gastrointestinal symptoms after liver transplantation for hereditary transthyretin amyloidosis

**DOI:** 10.1186/s12876-015-0284-4

**Published:** 2015-04-25

**Authors:** Jonas Wixner, Torbjörn Sundström, Pontus Karling, Intissar Anan, Ole B Suhr

**Affiliations:** 1 Departments of Public Health and Clinical Medicine, Umeå University, S-90187 Umeå, Sweden; 2 Radiation Sciences, Umeå University, S-90187 Umeå, Sweden

**Keywords:** Amyloidosis, Hereditary, Functional gastrointestinal disorders, Gastric emptying, Transplantation, Liver, Transthyretin

## Abstract

**Background:**

Hereditary transthyretin amyloid (ATTR) amyloidosis is a rare but fatal autosomal dominant condition that is present all over the world. A liver transplantation has been shown to halt the progress of the disease in selected patients and is currently considered to be the standard treatment. Gastrointestinal manifestations are common in hereditary ATTR amyloidosis and are important for the patients’ morbidity and mortality. The aim of this study was to evaluate the long-term outcome of gastric emptying, gastrointestinal symptoms and nutritional status after liver transplantation for the disease.

**Methods:**

Swedish patients with hereditary ATTR amyloidosis transplanted between 1990 and 2012 were included. A standardized method for measuring gastric emptying with a Tc^99m^-labelled meal followed by scintigraphy was utilized. Validated questionnaires were used to assess gastrointestinal symptoms and the modified body mass index (mBMI), in which BMI is multiplied by s-albumin, was used to evaluate nutritional status. Non-parametrical statistical tests were used.

**Results:**

Gastric emptying rates and nutritional statuses were evaluated approximately eight months before and two and five years after liver transplantation, whereas gastrointestinal symptoms were assessed in median nine months before and two and nine years after transplantation. No significant change was found in gastric emptying (median half-time 137 vs. 132 vs. 125 min, p = 0.52) or nutritional status (median mBMI 975 vs. 991 vs. 973, p = 0.75) after transplantation. Gastrointestinal symptom scores, however, had increased significantly over time (median score 7 vs. 10 vs. 13, p < 0.01).

**Conclusions:**

Gastric emptying and nutritional status were maintained after liver transplantation for hereditary ATTR amyloidosis, although gastrointestinal symptom scores had increased over time.

**Electronic supplementary material:**

The online version of this article (doi:10.1186/s12876-015-0284-4) contains supplementary material, which is available to authorized users.

## Background

Hereditary transthyretin amyloid (ATTR) amyloidosis or familial amyloid polyneuropathy (FAP) is a rare autosomal dominant disease caused by mutated transthyretin (TTR). The disease is present all over the world with endemic areas in Sweden, Portugal, Brazil and Japan [1].

The amyloidogenic TTR mutations decrease the stability of the TTR tetramer, which facilitates separation into misfolded monomers that, in turn, assemble into beta structured fibrils that build up the extracellular amyloid deposits [2]. The amyloid deposits elicit structural and toxic effects on the surrounding tissues [3-5] and peripheral and autonomic neuropathies, cardiomyopathy, cardiac arrhythmias and gastrointestinal (GI) disturbances are common complications of the disease.

GI disturbances play an important role in the morbidity and mortality of patients with hereditary ATTR amyloidosis [6,7], and virtually all Swedish patients develop GI complications during the course of the disease [8]. Initial symptoms are often constipation and/or nausea and vomiting. The constipation is later relieved by bursts of diarrhea that successively become continuous and fecal incontinence and severe malnutrition are common in later stages of the disease [9].

A liver transplantation (LTx) ceases the synthesis of mutated TTR and has, in selected patients, been proven to halt the progression of the disease [10,11]. Some studies have even demonstrated a clinical improvement after LTx [12,13], but most studies report unchanged or increased disease manifestations after the procedure [14-18]. Previous studies from our centre have demonstrated a largely unchanged GI function after transplantation [19,20].

The aim of the present study was to re-evaluate the long-term outcome of gastric emptying, GI symptoms and nutritional status after LTx for hereditary ATTR amyloidosis, based on data from the Swedish patient material collected over more than 20 years.

## Methods

### Patients

Symptomatic patients with hereditary ATTR amyloidosis who had been examined at Norrlands University Hospital, Umeå, Sweden and had undergone LTx as of January 2012 were included in the study. The liver transplantations had been performed at the transplantation centers at Karolinska University Hospital, Stockholm or Sahlgrenska University Hospital, Gothenburg, Sweden.

All patients had biopsy proven amyloid deposits and TTR mutations determined by DNA sequencing. Patients with an age at onset of less than 50 years were defined as early-onset cases. The data analyzed in the study, except for questionnaire data, were obtained from routine clinical investigations performed for evaluation of the disease both before and after LTx.

### Gastric Emptying Scintigraphy (GES)

Gastric emptying was measured according to the method employed in the Swedish multi-center study of gastric emptying [21]. The scintigraphic acquisitions were performed using the STARCAM and Millennium MPR gamma cameras (General Electric, Milwaukee, WI), both with a low energy, general-purpose collimator and a 128 x 128 matrix. The software used for the scintigraphic data calculations was updated in April 2006 and thereby also integrated in the main software connected to the modality. In connection with the software update, the number of data points was reduced as the time interval between the recordings was extended from approximately 10 to 30 min. The scintigraphic acquisition procedure, however, remained unchanged.

**Figure 1:**
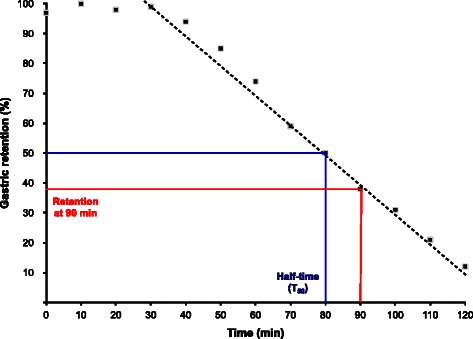
Gastric emptying scintigraphy. Graph showing the result of a normal gastric emptying scintigraphy and the variables used in the study. Lag phase from 0 to 30 min, a T_50_ of 80 min and a retention at 90 min of approximately 38%.

Two variables were used to assess gastric emptying rates – total half-time (T_50_) and retention (%) at 90 min (Figure 1). Since the scintigraphic software had changed during the study period, the T_50_ values were manually measured for consistency over time [22]. The retention at 90 min was added for verification of the results, and it was electronically calculated from the curves generated by both the old and the new software.

Delayed gastric emptying was defined as T_50_ above 133 minutes (mean + 2 SD) or a gastric retention of more than 76% (mean + 2 SD) at 90 min, according to the reference values obtained by the Swedish multi-center study of 160 healthy individuals [21]. T_50_ values over 350 min were entered as 350 min.

### Questionnaires

Two different questionnaires were used in the study and consent to participate was considered to be provided if the patient had completed and returned the questionnaires by mail.

The first questionnaire (see Additional files 1 and 2) contained seven items on GI symptoms, of which six were combined into two symptom clusters – upper or lower GI symptoms. Nausea, vomiting and loss of appetite were regarded as upper GI symptoms, whereas constipation, diarrhea and fecal incontinence were regarded as lower GI symptoms. The seventh item, loss of weight, was not included in the symptom clusters. A ten-point rating scale (0–10) was used for symptom assessment, giving a maximum possible score of 70, and the symptom scores were clustered into five levels – 0 (no symptoms), 1–3 (mild symptoms), 4–6 (considerable symptoms), 7–9 (severe symptoms) and 10 (unbearable symptoms). This questionnaire was utilized at all three time-points in the current study, and has previously been validated and used in other studies on Swedish patients with hereditary ATTR amyloidosis [15,23].

The second questionnaire contained four questions regarding concomitant diseases, current medication and consent to access the patients’ medical records for reviewing other factors with possible impact on their GI function. The patients’ medication was recorded and poly-pharmacy was defined as five concomitant drugs or more [24,25].

### Nutritional status

The patients’ nutritional status was assessed by the modified body mass index (mBMI), in which BMI (kg/m^2^) was multiplied by serum albumin (g/L) to compensate for edema. Values below 750 were regarded as consistent with underweight and values below 600 were regarded as consistent with severe malnutrition [6,7].

### Statistical analysis

Non-parametrical tests were used for all analyses. Differences between groups were analyzed with the chi^2^ and the Mann–Whitney U Tests. Changes over time were analyzed with Friedman’s Two-Way Analysis of Variance by Ranks, Cochran’s Q test and the Wilcoxon Signed Rank Test. Data shown are medians (min-max). P values below 0.05 were regarded as statistically significant. IBM SPSS Statistics 20 and 22 for Macintosh were used for the analyses.

### Ethics

The Regional Ethics Board in Umeå, Sweden approved the study; reference number 2011-365-31 M.

## Results

### Patients

One hundred and fifteen patients had undergone LTx from November 1990 to September 2011. All patients but three carried the TTR V30M mutation, the other variants being the TTR L55Q, A97S and F33L mutations.

Twenty-one patients had died during the study period, in median 3.0 (0.0-14.6) years after transplantation. The deceased patients had a significantly higher median age at onset (56.5 vs. 45.5 years, p < 0.01) and at LTx (58.7 vs. 49.1 years, p < 0.01) than those who had survived, but no differences were found for gender (61.9 % vs. 58.5 % males, p = 0.78) or median disease duration at LTx (3.3 vs. 3.3 years, p = 0.56). Virtually all of the deceased patients had completed the evaluations before transplantation and nearly half of them had also completed the first follow-up after LTx, whereas only a few had carried out a second follow-up after LTx. All available data from the deceased patients were included in the analyses of GI function.

### GES

**Figure 2:**
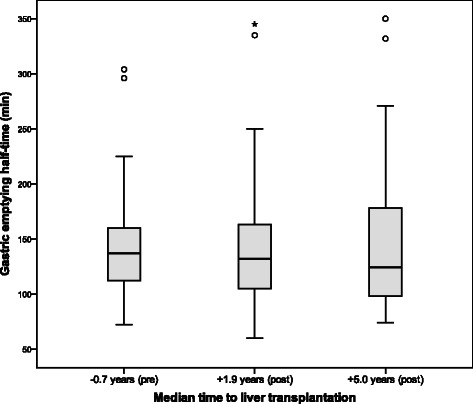
Outcome of gastric emptying after liver transplantation. Gastric emptying was scintigraphically measured using a Tc^99m^-labelled meal and the total half-time of the radioactive marker was used for the analyses. No significant change in gastric emptying half-times was found over time (p = 0.52). Only patients who had completed the scintigraphy at all three time-points were included (n = 26). P < 0.05 was regarded as statistically significant.

Ninety-nine patients (86.1%) had completed a GES prior to LTx, 71 patients (61.7%) at the first follow-up after LTx and 31 patients (27.0%) at the second follow-up. The overall outcome of the patients’ gastric emptying after LTx is presented in Figure 2 and no significant variation was found over time. A similar outcome was found for the retention at 90 min (median retention 78.1% vs. 77.8% vs. 78.6%, p = 0.61) and also for the fraction of patients with a delayed gastric emptying (50.0% vs. 46.2% vs. 42.3%, p = 0.79).

To investigate a possible deterioration while waiting for LTx, data from the pre-LTx evaluation and the initial assessment after LTx were separately analyzed, however, no significant change in gastric emptying was found between the assessments (median T_50_ 119 vs. 129 min, n = 67, p = 0.24).

### Questionnaires

The study questionnaires were sent by mail to the 92 patients (80.0%) who were still alive and Swedish residents at the time of the study and, of those, 77 (83.7%) had responded the questionnaires by June 2012. Six patients (7.8%) had completed the questionnaires less than two years after transplantation and their data were excluded from the outcome analyses of GI symptoms. Detailed pre-transplant characteristics of the patients are outlined in Table 1 and, overall, no significant differences were found between the patients who had responded the questionnaires and those who had not. Equivalent results were found after transplantation; however, at the second post-transplant follow-up, the number of patients was too small for adequate comparisons of gastric emptying half-times between the groups.

**Table 1:** **Patient characteristics**

	Responded study questionnaires		p value
	Yes	No	
	(n = 77)	(n = 15)	
Gender (males)	55.8%	66.7%	0.44
Late onset (≥50 years)	39.0%	26.7%	0.37
TTR V30M mutation	98.7%	93.3%	0.19
Age at onset (years)	47.3 (24.1-66.0)	39.5 (22.1-65.5)	0.07
Age at LTx (years)	50.1 (29.6-69.2)	42.4 (25.2-67.9)	0.08
Disease duration at LTx (years)	3.3 (0.5-12.1)	3.1 (1.9-9.0)	0.61
T_50_ pre LTx (min)	118 (48–350)	122 (56–235)	0.81
Total GI symptom score pre LTx	7 (0–35)	5.5 (0–21)	0.39
mBMI pre LTx	993 (550–1447)	973 (796–1320)	0.67
Age at study start (years)	58.9 (35.1-75.6)	53.3 (41.7-75.5)	0.12

Data shown are medians (min-max) and p < 0.05 was regarded as statistically significant. LTx: liver transplantation, mBMI: modified body mass index, n: number of subjects, T_50_: gastric emptying half-time, TTR: transthyretin.

Seventy (98.6%) of the patients who had filled out valid study questionnaires had completed corresponding questionnaires prior to their LTx and 59 patients (83.1%) had completed equivalent questionnaires approximately two years after transplantation. Hence, 59 patients had responded all three sets of questionnaires, which were completed in median 0.7 (0.1-1.6) years before LTx and 2.0 (1.0-5.9) and 8.7 (2.3-21.3) years after LTx, respectively.

### GI symptoms

**Figure 3:**
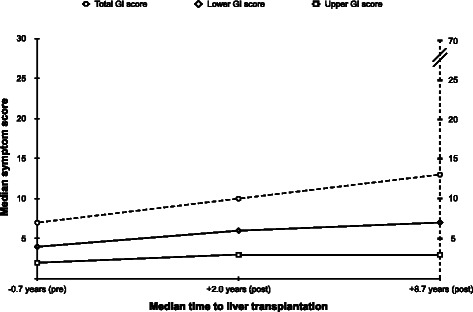
Outcome of gastrointestinal symptoms after liver transplantation. The total and lower gastrointestinal (GI) symptom scores had both increased significantly over time (p < 0.01, for both), whereas no significant change was found for upper GI symptom scores (p = 0.09). Nausea, vomiting and loss of appetite were regarded as upper GI symptoms, whereas constipation, diarrhea and fecal incontinence were regarded as lower GI symptoms. Unintentional weight loss was added to the total GI symptom category. All symptoms were evaluated with a 10-point rating scale and, thus, the maximum possible score was 70 for the total GI symptom category (dashed y axis), while only 30 for both the upper and lower GI symptom categories (solid y axis). Only patients who had completed all three sets of questionnaires were included (n = 57). P < 0.05 was regarded as statistically significant.

The outcomes of the GI symptom scores are displayed in Figure 3 and, overall, the symptom scores had increased after transplantation, as had the prevalence of GI complaints (84.2% vs. 91.2% vs. 96.5%, p = 0.03). As for the upper GI symptoms, there was no significant change in weight loss scores over time (median score 0 vs. 0 vs. 0, p = 0.71).

A significant rise in the total GI symptom score was found both between the pre-transplant evaluation and the initial assessment after LTx (median total GI score 6 vs. 9, n = 67, p = 0.03) and between the two post-transplant evaluations (median total GI score 9 vs. 12, n = 57, p = 0.02). Equivalent results were found for lower GI symptoms, whereas no significant change was found, at any time-point, for upper GI symptoms.

Subgroup analyses of the lower GI symptoms showed that the diarrhea scores (median score 0 vs. 2 vs. 3, p < 0.01) and fecal incontinence scores (median score 0 vs. 1 vs. 1, p < 0.01) had increased significantly over time, whereas no significant variation was found for constipation (median score 2 vs. 1 vs. 2, p = 0.17).

### Nutritional status

Data on nutritional status were available for 114 patients prior to transplantation, for 79 patients at first follow-up after LTx and for 41 patients at the last follow-up after LTx. The assessments were made in median 0.7 (0.1-1.8) years before LTx and 1.9 (0.9-4.6) and 4.7 (2.0-12.0) years after LTx, respectively, and no significant change in nutritional status was found over time (median mBMI 975 vs. 991 vs. 973, n = 33, p = 0.75). Moreover, no significant alteration was found between the pre-transplant and the initial post-transplant evaluation (median mBMI 971 vs. 941, n = 78, p = 0.38).

### Age at onset and gender

Subgroup analyses showed similar outcomes of gastric emptying, GI symptoms and nutritional status after LTx in early- and late-onset cases, as well as in males and females. However, in early-onset cases, no significant change in total GI symptom scores was found after transplantation (Table 2).

**Table 2:** **Outcome of gastrointestinal function in relation to age at onset and gender**

Subgroup	Variable	n	Pre LTx	1^st^post LTx	2^nd^post LTx	p value
Early-onset	T_50_ (min)	20	138	145	116	0.20
	Total GI symptom score	35	11	11	15	0.07
	mBMI	25	949	948	941	0.73
Late-onset	T_50_ (min)	6	130	106	163	0.57
	Total GI symptom score	22	4	6.5	9.5	<0.01
	mBMI	8	1006	1054	978	0.20
Male	T_50_ (min)	13	145	150	131	0.16
	Total GI symptom score	26	4	10.5	11.5	0.02
	mBMI	21	1028	1013	941	0.12
Female	T_50_ (min)	13	129	126	109	0.69
	Total GI symptom score	31	9	9	15	0.02
	mBMI	12	955	970	978	0.34

Only patients who had completed all three evaluations were included. Data shown are medians and p < 0.05 was regarded as statistically significant. Early-onset: <50 years of age, LTx: liver transplantation, mBMI: modified body mass index, T_50_: gastric emptying half-time.

Furthermore, early-onset cases reported significantly higher total GI symptom scores before LTx (median score 8 vs. 3.5, p < 0.01) and at the first follow-up after LTx (median score 11 vs. 6, p = 0.02) than late-onset cases. Female patients also reported higher total GI symptom scores (median score 7.5 vs. 4.5, p = 0.04) and had a significantly lower nutritional status (median mBMI 923 vs. 1016, p < 0.01) than males prior to transplantation.

### Medications

All patients received immunosuppressive therapy, most often tacrolimus (75.3%) and steroids (70.1%). Two patients were treated with insulin due to diabetes mellitus, whereas none received oral anti-diabetic agents. One patient received diflunisal [26] for his amyloidosis, but no patient was treated with tafamidis [27].

Differences in GI symptoms in relation to current medication were analyzed for the 15 (out of totally 32) drugs taken by ten individuals or more. Significant differences were found for four of the drugs (Table 3).

**Table 3:** **Relationship between medication and gastrointestinal symptoms**

Drug		n	Upper GI score	Lower GI score	Total GI score
Loperamide	Yes	12	4	14**	23**
	No	55	2	6	10
Paracetamol	Yes	15	6	13*	18**
	No	62	2	6	9
Anticonvulsants^a^	Yes	22	3	8.5*	14
	No	55	3	6	11
Beta blockers	Yes	18	7**	9	17.5
	No	59	2	6	11

Nausea, vomiting and loss of appetite were regarded as upper gastrointestinal (GI) symptoms, whereas constipation, diarrhea and fecal incontinence were regarded as lower GI symptoms. Unintentional weight loss was added to the total GI score. Medications with n < 10 were excluded. Data shown are medians and p < 0.05 was regarded as statistically significant. ^a^used against neuropathic pain, *p <0.05, **p <0.01.

Poly-pharmacy was observed in 45 (58.4%) of the patients who had responded the study questionnaires. No significant difference in total GI symptom scores (median score 11.5 vs. 12, p = 0.39) was found between the patients who had reported poly-pharmacy and those who had not.

## Discussion

The current study presents data on the outcome of the GI function after LTx for hereditary ATTR amyloidosis and is based on a large Swedish patient material collected over more than 20 years. Previous studies have, to the best of our knowledge, been based on smaller patient numbers and shorter follow-up times [19,20,28-31]. Most data used in the study were from routine clinical investigations; however, questionnaire data from the latest follow-up after LTx were collected specifically for the study. A questionnaire response rate of more than 80% supports the reliability of these results and, in addition, no significant differences in patient characteristics or GI function were found between patients who had responded the questionnaires and those who had not. A majority of the patients reported at least one GI symptom prior to LTx and virtually all patients (97%) had GI complaints at the last follow-up almost nine years after the procedure. Unexpectedly, since most previous studies have demonstrated a stable GI function [19,20,31], we found a significant increase both in symptom prevalence and symptom scores after transplantation. However, the symptom scores were generally low and only the lower GI symptom scores (i.e. the diarrhea and fecal incontinence scores) exhibited a significant increase over time. Moreover, the patients’ overall GI function seemed to be preserved as their gastric emptying rates, as well as their nutritional statuses, were maintained at follow-up in median five years after LTx. It is difficult to determine whether the increased GI symptoms were caused by a progression of the ATTR amyloidosis since GI complications, especially diarrhea, are common after LTx [32,33] and since GI symptoms are common side effects of several drugs. In attempt to answer this question, we analyzed the impact of the patients’ medication on GI symptom scores and significant differences were found for some of the drugs; however, none of them directly related to the LTx. It should be noted that loperamide was used to treat diarrhea, and its relationship with GI symptom scores should not be interpreted as loperamide being the cause of the intestinal symptoms, but the opposite. Furthermore, patients with more than five concomitant medications did not report higher GI symptom scores than those with less than five drugs, indicating that the patients’ medication is not the main factor behind their gastrointestinal complaints after LTx.

Despite that most patients received tacrolimus and steroids as immunosuppressive therapy, which both are associated with a risk of de-novo diabetes after transplantation [34,35], only two patients received treatment against diabetes at the time of the study. This suggests that diabetes mellitus is rare among patients with hereditary ATTR amyloidosis and also that a diabetic gastroenteropathy is not the cause of their increased GI symptom scores after LTx. Aging also has an effect on the function of the GI tract, especially with regard to the development of constipation and fecal incontinence [36,37]. Thus, the reported increase in fecal incontinence scores might be related to aging of the patients, however, the parallel increase in diarrhea scores is perhaps a more plausible explanation.

Altogether, the slight increase in GI symptom scores after LTx is most likely multi-factorial, but post-operative complications and medications probably contribute. Deterioration of the ATTR amyloidosis, however, appears to be a less likely cause since the objective measures of the GI function (i.e. gastric emptying half-time and nutritional status) were maintained. Additionally, we found no evidence of deterioration in GI function during the approximately eight month waiting time for transplantation. Since phenotypic differences have been observed between patients with early and late disease onset, subgroup analyses addressing this question were carried out. We found that early-onset cases reported significantly higher GI symptom scores before LTx and at the first follow-up after LTx, which is consistent with a more frequently occurring autonomic neuropathy in these patients [38,39]. Similar outcomes of gastric emptying and nutritional status were found for early- and late-onset cases, however, an increase in GI symptom scores was predominantly found in late-onset cases, who generally also have a poorer outcome after LTx [40]. Subgroup analyses were also performed with regard to gender since functional GI disorders generally are more common in females [41,42]. Indeed, female patients reported significantly higher GI symptom scores and a lower nutritional status prior to LTx, but no major gender related differences were found following transplantation.

### Limitations

In individuals with gastric retention, extrapolation is usually required to calculate gastric emptying half-times and the result may, in these cases, be less accurate than measurements based on percentages of retention at fixed time points [43]. Since a 2-hour scintigraphic measurement is the standard procedure at our hospital we used both of these methods to evaluate gastric emptying rates in the study.

The symptom scores used are depending on patients’ ability to correctly describe and grade their GI disturbances. To minimize errors, the same validated questionnaire was used throughout the study and patients were asked to state their current symptoms.

Since patients living outside the northern counties in Sweden are not routinely referred to our department for evaluation, attrition is a problem, especially between the first and second post-transplant follow-ups, and may give a selection bias towards patients with a favorable outcome after transplantation. In addition, the small remaining number of patients increases the risk of a type 2 statistical error, i.e. missing deterioration of gastric emptying after LTx.

## Conclusions

Gastric emptying rates and nutritional statuses appeared to be maintained in Swedish patients who had undergone LTx for hereditary ATTR amyloidosis, although their GI symptom scores had increased over time. GI symptom scores were higher in early-onset cases and in females; however, no major differences in the outcome of GI function after LTx were found in these subgroups of patients.

## Additional files

Additional file 1:
**Symptom questionnaire in Swedish.**
Additional file 2:
**Symptom questionnaire in English.**

## Abbreviations

A97S: Alanine substituted for serine at gene position 97
ATTR: Transthyretin amyloid
BMI: Body mass index
DNA: Deoxyribonucleic acid
F33L: Phenylalanine substituted for leucine at gene position 33
FAP: Familial amyloid polyneuropathy
GES: Gastric emptying scintigraphy
GI: Gastrointestinal
L55Q: Leucine substituted for glutamine at gene position 55
LTx: Liver transplantation
mBMI: Modified body mass index
SD: Standard deviation
T_50_: Total half-time
TTR: Transthyretin
V30M: Valine substituted for methionine at gene position 30
