# Supplementary material for: Outcome of gastric emptying and gastrointestinal symptoms after liver transplantation for hereditary transthyretin amyloidosis
Source: BMC Gastroenterol. 2015 Apr 25;15:51. doi: 10.1186/s12876-015-0284-4 (PMC4415350; doi:10.1186/s12876-015-0284-4)
Supplement: Additional file 1: — Symptom questionnaire in Swedish. [file 12876_2015_284_MOESM1_ESM.pdf]

Datum ______________

FRÅGOR OM SKELLEFTESJUKAN (FAP)

Markera genom att fylla i den siffra Du tycker motsvarar graden av Dina besvär just nu.

0	1, 2, 3	4, 5, 6	7, 8, 9	10
Inte alls	Ganska ringa<br>Lite	Betydande	Stark<br>Påtaglig<br>Mycket	Outhärdlig

Kroppssymptom

Illamående _________
Kräkningar _________
Diarré _________
Förstoppning _________
Problem att hålla avföringen _________
Matleda _________
Viktminskning _________
Yrsel _________
Svimningsbesvär _________
Allmän trötthets- eller matthetskänsla _________
Blåstömningsbesvär _________
Impotens _________
Synbesvär/ögonbesvär _________
Smärta i armar _________
Smärta i ben _________
Känselbortfall i armar _________
Känselbortfall i ben _________
Nedsatt muskelkraft i armar _________
Nedsatt muskelkraft i ben _________
Gångsvårigheter _________
Gripsvårigheter _________

Är det något som fattas i symptombilden som Du besväras av?
Ange i så fall vad och i vilken grad: _________

____

____

Markera genom att fylla i den siffra Du tycker motsvarar graden av Dina besvär just nu.

0	1, 2, 3	4, 5, 6	7, 8, 9	10
Inte alls	Ganska ringa<br>Lite	Betydande	Stark<br>Påtaglig<br>Mycket	Outhärdlig

Psykiska symptom

Oro/ångest ________
Stress/spänning ________
Rastlöshet ________
Irritation ________
Oföretagsamhet ________
Nedstämdhet ________
Likgiltighet ________
Livsleda ________
Sömnsvårigheter ________
Försämrad uppmärksamhet ________
Försämrad koncentration ________
Försämrat minne ________
Försämrad bedömningsförmåga ________
Kommunikationsbesvär ________

Är det något som fattas i symptombilden som Du besväras av?
Ange i så fall vad och i vilken grad: ________

____

____
