# Supplementary material for: Outcome of gastric emptying and gastrointestinal symptoms after liver transplantation for hereditary transthyretin amyloidosis
Source: BMC Gastroenterol. 2015 Apr 25;15:51. doi: 10.1186/s12876-015-0284-4 (PMC4415350; doi:10.1186/s12876-015-0284-4)
Supplement: Additional file 2: — Symptom questionnaire in English. [file 12876_2015_284_MOESM2_ESM.pdf]

Date _______________

FAP SYMPTOM QUESTIONNAIRE

Answer each item by filling out the figure that best matches your current disease state.

0	1, 2, 3	4, 5, 6	7, 8, 9	10
No symptoms	Mild symptoms	Considerable symptoms	Severe symptoms	Unbearable symptoms

Bodily symptoms

Nausea _________
Vomiting _________
Diarrhea _________
Constipation _________
Accidental bowel leakage _________
Loss of appetite _________
Weight loss _________
Dizziness _________
Fainting _________
Fatigue _________
Bladder emptying problems _________
Impotence _________
Visual disturbances _________
Arm pain _________
Leg pain _________
Sensory loss in the arms _________
Sensory loss in the legs _________
Reduced arm muscle strength _________
Reduced leg muscle strength _________
Difficulty walking _________
Difficulty gripping _________

Do you suffer from any additional symptom?
If so, please state and grade the symptom here: _________

____

____

Answer each item by filling out the figure that best matches your current disease state.

0	1, 2, 3	4, 5, 6	7, 8, 9	10
No symptoms	Mild symptoms	Considerable symptoms	Severe symptoms	Unbearable symptoms

Mental symptoms

Anxiety ________
Stress/tension ________
Restlessness ________
Irritability ________
Shiftlessness ________
Depression ________
Indifference ________
Tiredness of life ________
Sleep disturbances ________
Attention problems ________
Concentration difficulties ________
Memory problems ________
Impaired judgement ________
Communication difficulties ________

Do you suffer from any additional symptom?
If so, please state and grade the symptom here: ________

____

____
